# What is the impact of a novel *DEPDC5* variant on an infant with focal epilepsy: a case report

**DOI:** 10.1186/s12887-022-03515-8

**Published:** 2022-07-30

**Authors:** Chunyu Gu, Xiaowei Lu, Jinhui Ma, Linjie Pu, Xiufang Zhi, Jianbo Shu, Dong Li, Chunquan Cai

**Affiliations:** 1 Tianjin Children’s Hospital (Children’s Hospital of Tianjin University), Tianjin, 300134 China; 2 Graduate College of Tianjin Medical University, Tianjin, 300070 China; 3 The Medical Department of Neurology, Tianjin Children’s Hospital, No. 238 Longyan Road, Beichen District, 300134 Tianjin, China; 4 Electroencephalogram Laboratory, Tianjin Children’s Hospital, Tianjin, 300134 China; 5 Tianjin Pediatric Research Institute, Tianjin, 300134 China; 6 Tianjin Key Laboratory of Birth Defects for Prevention and Treatment, Tianjin, 300134 China; 7 Department of Neurosurgery, Tianjin Children’s Hospital, No. 238 Longyan Road, Beichen District, 300134 Tianjin, China

**Keywords:** *DEPDC5*, Focal epilepsy, Whole exome sequencing, FFEVF, Case report

## Abstract

**Background:**

Variants in the *DEPDC5* have been proved to be main cause of not only various dominant familial focal epilepsies, but also sporadic focal epilepsies. In the present study, a novel variant in *DEPDC5* was detected in the patient with focal epilepsy and his healthy father. We aimed to analyze the pathogenic *DEPDC5* variant in the small family of three.

**Case presentation:**

A 5-month-old male infant presented with focal epilepsy. Whole exome sequencing identified a novel heterozygous variant c.1696delC (p.Gln566fs) in *DEPDC5*, confirmed by Sanger sequencing. The variant was inherited from healthy father.

**Conclusions:**

Our study expands the spectrum of *DEPDC5* variants. Moreover, We discuss the relation between the low penetrance of *DEPDC5* and the relatively high morbidity rate of *DEPDC5*-related sporadic focal epilepsy. Besides, due to interfamilial phenotypic and genetic heterogeneity, we speculate the prevalence of familial focal epilepsy with variable foci might be underestimated in such small families. We emphasize the importance of gene detection in patients with sporadic epilepsy of unknown etiology, as well as their family members. It can identify causative mutations, thus providing help to clinicians in making a definitive diagnosis.

## Background

The *DEPDC5* (OMIM #614,191) is located on chromosome 22q12.2–12.3. It contains 43 exomes and encodes the disheveled, Egl-10 and pleckstrin domain-containing protein 5 (DEPDC5), a full-length protein composed of 1603 amino acids [1]. DEPDC5 is a part of the GAP Activity Toward Rags 1 complex, which also contains nitrogen permease regulator-like-2 and nitrogen permease regulator-like-3. They work together and regulate mammalian target of rapamycin complex 1 (mTORC1) negatively [2]. The mTORC1 participates in the regulation of neuronal growth, homeostasis and metabolism in neurons. Besides, mTORC1 plays an important role in the development of neurons, such as the differentiation of neurons, synapse formation and neurite outgrowth [3].

To date, variants in the *DEPDC5* have been proved to be main cause of various dominant familial focal epilepsies, such as autosomal dominant sleep-related hypermotor epilepsy, familial focal epilepsy with variable foci (FFEVF), familial temporal lobe epilepsy and focal epilepsy caused by various cortical developmental malformations [4]. According to the reports, 13% of familial lateral temporal lobe epilepsies caused by *DEPDC5* variants [5]. The *DEPDC5* is also related with 13% of autosomal dominant sleep-related hypermotor epilepsies [6]. What’s more, more than 80% FFEVF patients were caused by *DEPDC5* variants [4]. Besides, variants in *DEPDC5* have been detected in many patients with non-familial focal epilepsies [7]. Here we describe a 5-month-old male infant with focal epilepsy, and discuss what role a novel *DEPDC5* variant plays in the small family of three.

## Case presentation

The male infant was born to healthy non-consanguineous parents. There was no discovered family history of epilepsy or other nervous system diseases. The patient was the only child of his family. He was born at term with no asphyxia after an uneventfully pregnancy. His weight and body length were within the normal range at birth. The neonatal period was unremarkable. And up to now, there has been no problem with the patient’s developmental milestones.

**Fig. 1:**
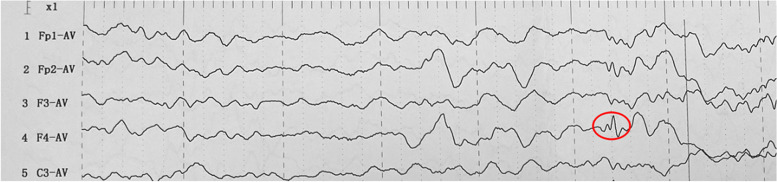
The abnormal EEG picture of proband. Epileptiform discharges with sharp wave (marked with a red circle) over the right frontal area were revealed. The amplitude was 93.8 μV and the timing was 82 ms

At the age of 5 months, the infant appeared the first afebrile epileptic seizure, which happened when he was sleeping. It lasted for 10 s approximately. And it was characterized by eyes blinking frequently, followed by binocular gaze and the tonic seizure of right upper limb. Asking for past medical history, we found it worth noting that the patient showed short-lasting symptom of blinking about half of a month ago. While, it failed to draw the attention of his parents. Physical examinations and the laboratory examinations showed no abnormality. The blood and urine metabolic screenings were normal as well. The brain magnetic resonance imaging (MRI) showed widened extracerebral space and widening of the beginning of left lateral fissure cistern. A video-electroencephalogram (EEG) revealed epileptiform discharges with spikes and sharp waves over the right frontal area (Fig. 1).

Before the infant received effective treatment, he experienced 9 times focal seizures with similar symptoms. The epileptic seizure usually happened before sleep or during sleeping period. In order to control the epileptic seizure, the patient received a treatment of phenobarbital (5 mg/kg) at first. However, the seizure wasn’t controlled until topiramate was applied. 5 months later, a follow-up review found that epileptic seizure had not occurred to the infant since he discharged from the hospital. And 6 months later, the re-examine of video-EEG showed nothing unusual. The psychomotor development didn’t show retardation or decline. So far, the patient has been seizure-free for 7 months.

**Fig. 2:**
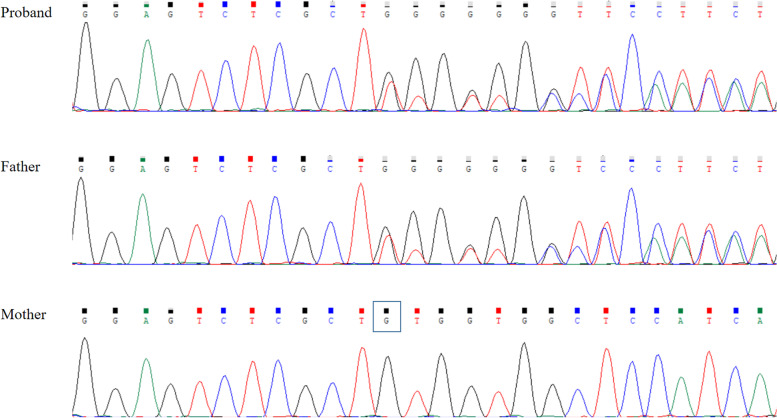
Sanger sequencing confirmed the *DEPDC5* (c. 1696delC) variant. Both the proband and his father father carried the heterozygous variant. The nucleotide deletion was marked with a dark box in the reverse sequencing

**Fig. 3:**
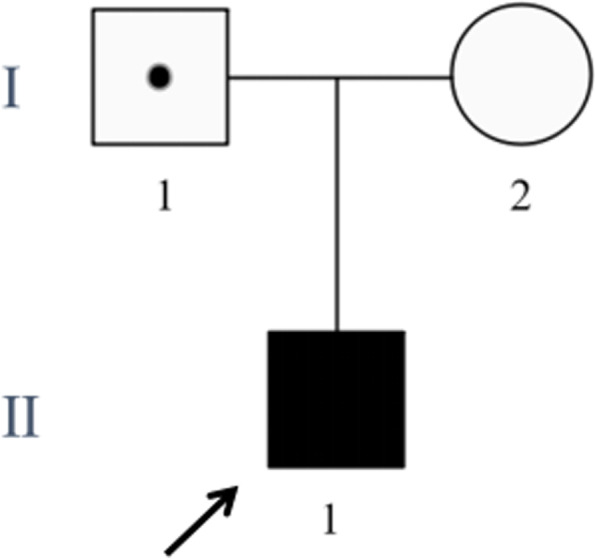
Pedigree chart in this family. The father (I1) was healthy (marked as a dark dot in a blank background) even though he carried the heterozygous variant c.1696delC in DEPDC5. The proband (II1, pointed out by the arrow) inherited the variant from his father and developed the corresponding disease (marked as dark)

We performed whole exome sequencing (WES) to the patient and detected a novel heterozygous variant in *DEPDC5* (NM_001242896.1), c.1696delC. The variant was autosomal dominant and could result in a frameshift in translation (p.Gln566fs) and a premature termination codon (PVS1). Besides, it was absent in healthy controls in the 1000 Genomes Project Database [8], the Exome Aggregation Consortium Database [9] or National Heart, Lung, and Blood Institute Exome Sequencing Project [10] (PM2). And it was not recorded in Human Gene Mutation Database [11]. As a result, the variant c.1696delC in *DEPDC5* was classified as “likely pathogenic” based on the above evidence according to the 2015 American College of Medical Genetics and Genomics variant classification guidance [12]. The variant was confirmed by Sanger sequencing (the primers used for amplifying were forward 5’—TCTTCAGGCAGTGTCCTTC—3’ and reverse 5’—AGCAACCAACTTACCCACA—3’) using the DNA of the family members (see Fig. 2). And the result revealed that the variant was inherited from healthy father. The mother didn’t carry this alteration. The pedigree chart in this family is shown in Fig. 3.

## Discussion and conclusions

*DEPDC5* variants have been demonstrated to be the most common cause of familial focal epilepsies. While in clinical work, most patients have no family history of epilepsy. In 2017, a study reported that 2 in 220 (0.9%) patients with sporadic non-lesiona focal epilepsy, had disease-causing variants in the *DEPDC5* [7]. It suggested *DEPDC5* also have a significant effect on sporadic focal epilepsy. FFEVF, proved mainly caused by *DEPDC5* variants, is a type of autosomal dominant epilepsy. It is characterized by a obvious intrafamilial variation that focal seizures happen in different cortical regions within the family members. Seizure type varies considerably between individuals, rather than showing phenotypic homogeneity [13]. Because of its interfamilial phenotypic and genetic heterogeneity, we speculate that the prevalence of FFEVF might be underestimated in small families. Interestingly, in the case we reported, the infant’s variant was inherited from his healthy father who carries the same variant as the infant. In addition to considering that variable expressivity could be caused by the combination of genetic, environmental, and lifestyle factors, this is accordant with the low penetrance (45–67%) which has been reported in previous studies [7]. Besides, viewing Table 1 which shows clinical expressions of various focal epilepsies caused by *DEPDC5* variants as well as that of proband, it is impossible to exclude the probability of FFEVF. Though this family is so small that we can’t diagnose the epilepsy as FFEVF clinically yet. In view of this situation, the misdiagnosed families may contribute to the relatively high morbidity rate of sporadic focal epilepsy. To some degree, it can explain why *DEPDC5* variants account for nearly 1% of patients with sporadic focal epilepsy. It suggests that gene detection is necessary for the patients with sporadic epilepsy of unknown etiology, as well as their family members. The identification of *DEPDC5* variants in these small families enables the possibility of molecular diagnosis with FFEVF. In the present study, WES played a critical role in the differentiation and diagnosis of disease. Considering the clinical and genetic heterogeneity of FFEVF, WES represents a rapid, cost-effective and accurate method for the diagnosis of the disease.

**Table 1:** Clinical expressions of various focal epilepsies caused by *DEPDC5* variants

	**Focal epilepsies (former nomenclature)**			
	**ADSHE **[6] **(ADNFLE)**	**ADEAF **[5] **(ADLTE)**	**FFEVF **[4]	**Proband’s** **epilepsy**
**Average age at onset**	Childhood (8 years-12 years)	Adolescence-Adult (16.24 years)	Infancy-Adult (3 months-40 years)	5 months
**Site of onset**	Frontal lobe	Lateral temporal lobe	Individuation, intrafamilial variation	Right frontal lobe
**Features of seizures**	Sleep-related motor seizures	With auditory auras and symptoms	Individuation, intrafamilial variation	Binocular gaze, tonic seizure of right upper limb
**Brain MRI**	Generally normal			Unremarkable
**EEG**	Frontal epileptiform discharges	Lateral temporal epileptiform discharges	Individuation, intrafamilial variation	Right frontal epileptiform discharges

*ADSHE* Autosomal dominant sleep-related hypermotor epilepsy, *ADNFLE* Autosomal dominant nocturnal frontal lobe epilepsy, *ADEAF* Autosomal dominant epilepsy with auditory features, *ADLTE* Autosomal dominant lateral temporal lobe epilepsy

Previous studies have generally believed that focal epilepsies caused by *DEPDC5* variants are non-lesional [13]. In recent years, many teams have found focal cortical dysplasia (FCD) in epilepsy patients through brain MRI and neuropathology [7, 14]. Most of them agreed that FCD results from a second-hit somatic variant [15]. It is explained that *DEPDC5* somatic variants trigger FCD through mammalian target of rapamycin hyperactivation in dysmorphic neurons [15]. Common MRI features of FCD include T2 hyperintensity of the white matter, abnormally deep sulcus, transmantle sign, cortical thickening, and blurring of the gray-white matter border [16]. In the case we reported, the abnormal brain MRI may be a common physiological manifestation in infants. Besides, the patient’s seizure is controlled well by topiramate monotherapy. While the patients with FCD usually presented frequent and refractory seizures. Therefore, it is unlikely that the patient will be complicated with FCD. In view of the abnormal MRI, a further observation is still needed. High resolution MRI can be considered to detect subtle cortical malformations. Moreover, Regular follow-up and the re-examination of EEG should be arranged.

In rat models, heterozygous *Depdc5*^±^ rats had altered cortical neuron excitability and firing patterns but without developmental abnormalities or spontaneous seizures. However, homozygous *Depdc5*^−/−^ embryos died in utero caused by global growth delay. The study revealed a potential quantitative correlation between genetic impairment and phenotype severity. And heterozygous variants would probably cause susceptibility alterations or mild phenotype [17]. Febrile seizures plus/febrile seizures and FCD was defined as phenotypes of *DEPDC5* variants, through evaluating evidence from five clinical-genetic aspects [18]. Further analysis revealed that FCD was more frequently associated with null variants. In contrast, febrile seizures plus/febrile seizures had a high frequency of missense variants. It was coincident with the findings from gene knockout rat models. Besides, the study proposed that variants closer to the binding site of *DEPDC5* to nitrogen permease regulator-like-2/nitrogen permease regulator-like-3 complex could lead to more severe phenotype like FCD.

In conclusion, we found a novel heterozygous variant in *DEPDC5,* c.1696delC (p.Gln566fs) in a family of three. The variant spectrum of *DEPDC5* is expanded. Due to interfamilial phenotypic and genetic heterogeneity, the prevalence of FFEVF might be underestimated in such small families. Considering the existence of an unaffected carrier, FFEVF is possible to be diagnosed clinically. In clinical practice, gene detection can provide support to a definitive diagnosis and it is necessary for the patients with sporadic epilepsy of unknown etiology, as well as their family members.

## Data Availability

The datasets used and/or analysed during the current study are available in ClinVar, accession number is SCV002025780.

## Abbreviations

DEPDC5: Disheveled, Egl-10 and pleckstrin domain-containing protein 5
mTORC1: Mammalian target of rapamycin complex 1
FFEVF: Familial focal epilepsy with variable foci
MRI: Magnetic resonance imaging
EEG: Electroencephalogram
WES: Whole exome sequencing
FCD: Focal cortical dysplasia
